# Characteristics of leptospirosis patients admitted to a tropical university hospital during the 2000 to 2010 period

**DOI:** 10.1186/cc10647

**Published:** 2012-03-20

**Authors:** H Mehdaoui, E Caffiot, R Theodose, R Valentino, D Resiere, C Chabartier, M Jonas

**Affiliations:** 1 Fort de France University Hospital, Fort De France, Martinique

## Introduction

Leptospirosis is an endemic disease in the intertropical area. Most of the patients present with mild to moderate clinical forms, but leptospirosis may lead to multiple organ failure and death.

## Methods

We retrospectively analyzed the characteristics of 113 patients with leptospirosis admitted to our emergency department.

## Results

PCR and/or immunological investigations confirmed the diagnosis for 88 patients. We compared the periods before and after PCR diagnosis implementation (2006), and determined the pattern of the most severe forms. Thirty-two patients were admitted to our ICU. Eight of the ICU patients died including four with confirmed diagnosis. Nineteen patients were diagnosed before 2006, and 69 during the period to 2010. Patients were less frequently admitted to the ICU during the second period (29% vs. 63%, *P *= 0.013). ICU patients had a higher heart rate (111 ± 28 vs. 93 ± 21, *P *= 0.001), and had more frequently jaundice (94% vs. 64%, *P *= 0.002) and oliguria (81% vs. 23%, *P *< 0.001). Glycemia (8.7 ± 3.3 vs. 7.1 ± 3.4, *P *= 0.04), creatinin (530 ± 299 vs. 142 ± 113, *P *< 0.0001), bilirubin (423 ± 251 vs. 69 ± 103, *P *< 0.0001), CRP (325 ± 135 vs. 210 ± 127, *P *< 0.0001), and WBCC (21.7 ± 9.5 vs. 9.7 ± 5.3, *P *< 0.0001) were higher and protidemia (58 ± 15 vs. 68 ± 13, *P *= 0.002), hematocrit (24 ± 6 vs. 34 ± 6, *P *< 0.0001), and P/F ratio (271 ± 127 vs. 352 ± 84, *P *= 0.036) were lower in the ICU group. Troponin was increased more frequently in the ICU group (44% vs. 9%, *P *= 0.0003) and ECG anomalies (78% vs. 52%, *P *= 0.02) were more frequent. Among the 22 early cardiac echographies performed in the ICU, 11 patients had LVEF <50%.

## Conclusion

The use of PCR dramatically improved the diagnosis of mild to moderate forms of leptospirosis and led to an apparent increase its incidence. Severe forms were less easy to assess as they occur later and we should have a more aggressive policy to improve the immunological diagnosis which was sometimes neglected since the implementation of PCR diagnosis. Severe forms have a more pronounced inflammatory syndrome and diffuse organ failure. Aggressive fluid loading as recommended in septic states may have worsened hemodynamic and respiratory conditions in the ICU group. This is suggested by the hemodilution pattern found in this group. The association of renal, myocardial and respiratory failures in leptospirosis should lead to a careful monitoring of fluid loading and myocardial status.

